# Metabolomics of Acute vs. Chronic Spinach Intake in an Apc–Mutant Genetic Background: Linoleate and Butanoate Metabolites Targeting HDAC Activity and IFN–γ Signaling

**DOI:** 10.3390/cells11030573

**Published:** 2022-02-07

**Authors:** Ying-Shiuan Chen, Jia Li, Sultan Neja, Sabeeta Kapoor, Jorge Enrique Tovar Perez, Chakrapani Tripathi, Rani Menon, Arul Jayaraman, Kyongbum Lee, Wan Mohaiza Dashwood, Shan Wang, Ke Zhang, Koichi Kobayashi, Praveen Rajendran, Roderick Dashwood

**Affiliations:** 1 Center for Epigenetics & Disease Prevention, Texas A&M Health, Houston, TX 77030, USA; vickychen0409@gmail.com (Y.-S.C.); sultanabda@tamu.edu (S.N.); sabeetak@tamu.edu (S.K.); jtovar@tamu.edu (J.E.T.P.); chakrapani010@tamu.edu (C.T.); wdashwood@tamu.edu (W.M.D.); kzhang@tamu.edu (K.Z.); prajendran@tamu.edu (P.R.); 2 Department of Clinical Cancer Prevention, The University of Texas MD Anderson Cancer Center, Houston, TX 77030, USA; 3 Department of Chemical Engineering, College of Engineering, Texas A&M University, College Station, TX 77840, USA; ranjm@tamu.edu (R.M.); arulj@tamu.edu (A.J.); 4 Department of Chemical and Biological Engineering, Tufts University, Medford, MA 02155, USA; Kyongbum.lee@tufts.edu; 5 Department of Biology and Biochemistry, University of Houston, Houston, TX 77204, USA; shanwillons@gmail.com; 6 Department of Immunology, Hokkaido University Graduate School of Medicine, Sapporo 060–8638, Japan; kskobayashi@med.hokudai.ac.jp or; 7 Department of Microbial Pathogenesis and Immunology, Texas A&M Health Science Center, Bryan, TX 77087, USA; 8 Department of Translational Medical Sciences, and Antibody & Biopharmaceutics Core, Texas A&M College of Medicine, Houston, TX 77030, USA

**Keywords:** Familial Adenomatous Polyposis, gut microbiome, histone deacetylase, interferon–γ signaling, major histocompatibility complex, polyposis in rat colon, spinach

## Abstract

There is growing interest in the crosstalk between the gut microbiome, host metabolomic features, and disease pathogenesis. The current investigation compared long–term (26 week) and acute (3 day) dietary spinach intake in a genetic model of colorectal cancer. Metabolomic analyses in the polyposis in rat colon (Pirc) model and in wild–type animals corroborated key contributions to anticancer outcomes by spinach–derived linoleate bioactives and a butanoate metabolite linked to increased α–diversity of the gut microbiome. Combining linoleate and butanoate metabolites in human colon cancer cells revealed enhanced apoptosis and reduced cell viability, paralleling the apoptosis induction in colon tumors from rats given long–term spinach treatment. Mechanistic studies in cell–based assays and in vivo implicated the linoleate and butanoate metabolites in targeting histone deacetylase (HDAC) activity and the interferon–γ (IFN–γ) signaling axis. Clinical translation of these findings to at–risk patients might provide valuable quality–of–life benefits by delaying surgical interventions and drug therapies with adverse side effects.

## 1. Introduction

Complex interrelationships govern the dynamic interactions between gut microbes, host metabolomics, and exogenous drivers of disease outcome [1,2,3,4]. Irritable bowel syndrome, obesity, metabolic disorders and malignancies linked to gut dysbiosis and altered microbial diversity might be ameliorated via novel biotherapies and bioactive food constituents [1,2,3,4]. Metabolomic approaches have been adopted in human intervention trials, as in the case of urinary biomarkers of spinach (SPI) intake associated with the health benefits of green leafy vegetables [5], but the methodologies are not well established in target tissues of the gastrointestinal tract or following systemic uptake.

We recently reported on a multi–omic investigation of cancer prevention by SPI in the polyposis in rat colon (Pirc) model [6]. The Pirc rat is analogous to Apc–mutant mouse models, such as Apc^Min/+^ mice, but more precisely mimics the small intestine and colon tumor burden observed in human hereditary Familial Adenomatous Polyposis (FAP) [7]. In the Pirc model, rats fed baby SPI for 26 weeks (10% *w*/*w*, freeze–dried in the diet) exhibited significant antitumor efficacy, with greater than 60% reduced tumor multiplicity in the colon and small intestine [6]. Increased gut microbiome diversity after SPI intake coincided with reversal of taxonomic composition. Metagenomic prediction implicated linoleate and butanoate metabolism, which was supported by untargeted metabolomics. Specifically, when colon tumors were compared with matched normal–looking tissues, anticancer outcomes were linked to SPI–derived linoleate bioactives with known pro–apoptotic/anti–inflammatory mechanisms, as well as altered butanoate metabolism stemming from increased α–diversity of the gut microbiome. The metabolomic study included rats that were given SPI (freeze–dried, 10% *w*/*w* in the diet) for only 3 days; the latter findings are presented here for the first time, yielding new insights into acute vs. chronic SPI intake and the associated immunoepigenetic mechanisms.

## 2. Materials and Methods

**Figure 1:**
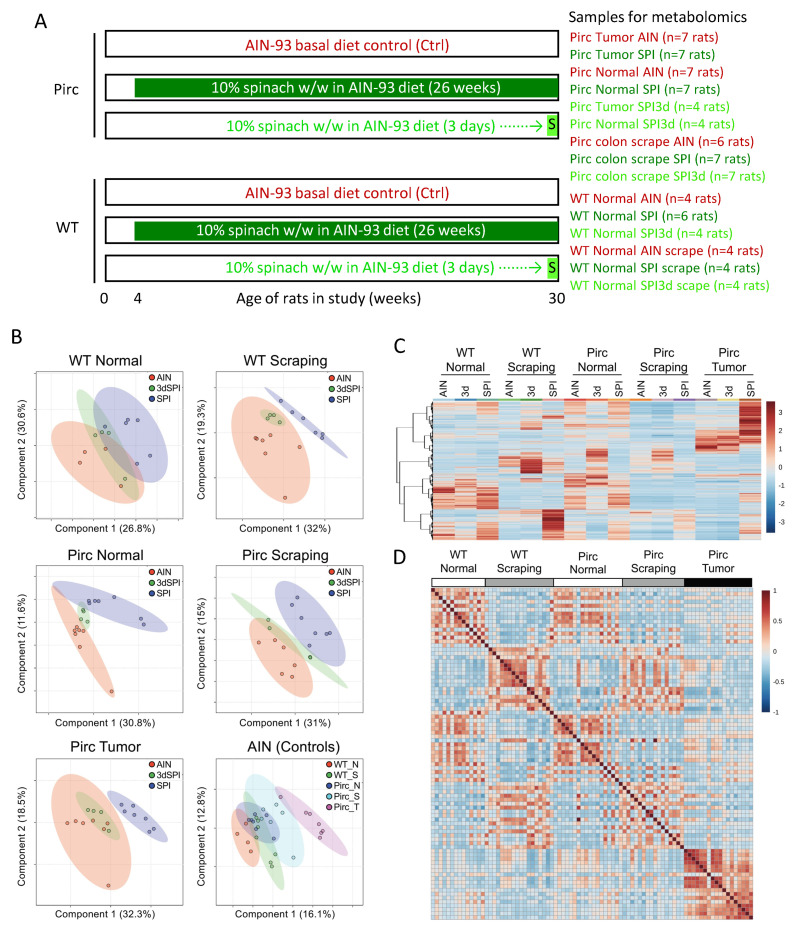
Dosing scheme and metabolomic analyses in Pirc and wild–type (WT) rats. (**A**) Animals were given AIN basal diet or AIN diet containing 10% spinach (*w*/*w*) for 26 weeks (SPI) or for 3 days (SPI3d). At necropsy, adenomatous colon polyps, normal colon ‘punch’ biopsies, and colonic mucosa scrapings were collected. (**B**) Partial least squares discriminant analysis of 17,243 metabolomic features. (**C**) Heatmap of 5946 hierarchically clustered significant features, representing the average of each group and auto–scaled by feature. (**D**) Correlation analysis across tissue categories.

Animals—Studies in Pirc (F344/NTac–*Apc^am1137^*) and wild–type (WT) F344 male rats were approved by the Institutional Animal Care and Use Committee. For complete details on preclinical methodologies refer to Chen et al. [6]. In brief, Pirc and WT rats were assigned randomly to basal AIN93 control diet or AIN93 diet containing 10% *w*/*w* freeze–dried baby SPI. Rats were fed SPI from 4 to 30 weeks of age (26–wk SPI intake), or for 3 days only (SPI3d), starting in the final week of the 30 week study. At necropsy, tissue sampling for metabolomic analyses included Pirc colon tumors, adjacent normal–looking colonic mucosa, colonic mucosa scrapings, colon ‘punch’ biopsies, and normal colon from WT rats, with biological replicates as indicated in Figure 1A (see Section 3.1).

Metabolomics—Pre–weighed samples of rat colon tumor and normal colonic mucosa, collected at the time of necropsy, were homogenized in 0.5 mL cold methanol and 0.2 mL chloroform in pre–cooled Garnet bead tubes using a Precellys^®^24 beadbeater (Zymo Research, Irvine, CA, USA). Samples were centrifuged at 3000 rpm for 10 min at 4 °C and 0.7 mL cold water was added to the supernatant. The aqueous phase was collected by centrifugation at 3000 rpm for 1 min and passed through a sterile nylon cell strainer and lyophilized. Samples were reconstituted in 50 μL methanol/water (1:1, *v*/*v*) and stored at −80 °C. Liquid chromatography high–resolution accurate–mass spectrometry was conducted as reported [6]. A Synergi Fusion–RP C–18 column (Phenomenex, Torrance, CA, USA) was used with a methanol/acetonitrile solvent gradient, and mass scanning in the positive mode was in the range 50 to 750. MS1 and MS1–dependent MS2 spectra were collected at an *m*/*z* resolution of 70,000 and 17,500, respectively, with the autosampler maintained at 4 °C. Raw metabolomic data were imported into Progenesis QI (Waters, Milford, MA, USA) for alignment, peak picking, and metabolite identification, with reference to the Human Metabolome Database (HMDB). Raw abundance data were normalized to initial sample weights, incorporating Partial Least Squares Discriminant Analysis (PLSDA). Features were filtered by their appearance in three independent metabolomic databases, with at least three biological replicates and a significant ANOVA test. Significant features were subjected to clustering and correlation by MetaboAnalyst 4.0. The *p*–values (two–tailed *t*-test) and *t*-scores (standardized test statistic) were generated for multiple group comparisons of metabolic networks and functional metabolite prediction via Mummichog version 2 in R. Compound names were mapped to Kyoto Encyclopedia of Genes and Genomes (KEGG) COMPOUND, and pathway analyses by Mummichog were ranked according to *p*–value, using *p* = 0.05 as the cutoff. For further information on the untargeted metabolomics, see Chen et al. [6].

Microbiome—Detailed methodologies were reported by Chen et al. [6]. In brief, rat fecal samples were submitted for bacterial genomic DNA extraction at the Center for Metagenomics & Microbiome Research (CMMR), Baylor College of Medicine, Houston, TX. The 16S rDNA V4 region was amplified and barcoded via PCR and sequenced using the MiSeq platform (Illumina, San Diego, CA, USA) with a 2 × 250 bp paired–end protocol. OTUs at a similarity cutoff value of 97% were generated by the UPARSE algorithm and mapped to SILVA database. OTU tables and Agile Toolkit for Incisive Microbial Analyses (ATIMA) were provided by CMMR for primary data visualization. ATIMA microbiome data were subjected to the Kruskal–Wallis test, as before [6].

Proteins—Immunoblotting was performed as reported [8,9,10,11,12,13,14,15]. Primary antibodies and concentrations were as follows (anti–): β–catenin #9581 1:1000, poly(ADP–ribose) polymerase (PARP) #9542s 1:1000, cleaved caspase–3 (CC3) #9661s 1:1000, c–Myc #D3N8F 1:1000, Cyclin D1 #2922s 1:1000, Survivin #1808s 1:1000 and nuclear factor of κ light polypeptide gene enhancer in B–cells inhibitor, α (IκBα) #9242 1:1000 (Cell Signaling, Danvers, MA, USA); matrix metalloproteinase–7 (Mmp7) #NB300–1000 1:500 and forkhead box P3 (Foxp3) #NBP2–41205 1:500 (Novusbio, Littleton, CO, USA); NLR Family CARD domain containing 5 (NLRC5) #PA5–21017 1:500 and beta 2–microglobulin (β2m) #PA5–88527 1:1000 (Invitrogen, Carlsbad, CA, USA); Transporter 1 ATP binding cassette subfamily member 1 (TAP1) #11114–1–AP 1:1000 (Proteintech, Rosemont, IL, USA); interferon–γ (IFN–γ) #A12450 1:1000 (ABclonal, Woburn, MA, USA); and β–actin #A1978 1:500 (Millipore Sigma, St. Louis, MO, USA). Secondary antibodies were goat–anti–mouse IgG #1706516 1:10,000 or goat–anti–rabbit IgG #1706515 1:10,000 (Bio–Rad, Hercules, CA, USA). Membranes were washed and incubated with horseradish peroxidase–conjugated secondary antibody for 1 h. Bands were visualized using Western Lightning Plus–ECL Enhanced Chemiluminescence Substrate (Perkin Elmer, Waltham, MA, USA) and detected using a ChemiDoc^MP^ Imaging System (Bio–Rad).

RNA—Real–time quantitative PCR (RT–qPCR) was conducted as before [14,15]. In brief, RNA was extracted from cell pellets using a NucleoSpin kit (Macherey–Nagel, Bethlehem, PA, USA), with quantification via a Cytation5 microplate reader (Thermo Fischer Scientific, Waltham, MA, USA). Reverse–transcription was performed utilizing SuperScript III (Thermo Fisher Scientific). Gene expression was quantified by qPCR in a 10–µL reaction consisting of cDNAs, SYBR green dye (Genesee Scientific, El Cajon, CA USA), and gene–specific primers, in a LightCycler 480 II (Roche, Indianapolis, IN, USA). Each sample was subjected to three independent experiments, and quantification was based on the Ct value. Gene–specific primers from DNA Technologies (Coralville, Iowa, USA) were as follows: *B2M:* 5′–TGTAAAGGGCCTCAGTGATTC–3′ (F), 5′–AGGAAAGAACGCTGGCTAAA–3′ (R); *IFNG:* 5′–GAGCTTTGATGAGCACCGAG–3′ (F), 5′–GCAGGCATCAGTCCAGTATTC–3′ (R). *Glyceraldehyde–3–Phosphate Dehydrogenase* (*GAPDH)* primers were as reported [15].

Cells—HCT116 human colon cancer cells from American Type Culture Collection (Manassas, VA, USA) were validated and used in viability, fluorescence–activated cell sorting (FACS), histone deacetylase (HDAC) activity, in silico, and immunoblotting assays, as reported [8,9,10,11,12,13,14,15,16,17]. Murine MC38 and MC38–OVA colon carcinoma cells [16], kindly provided by Dr. Arlene Sharpe (Harvard Medical School, Boston, MA, USA), also were treated with test agents for 7 d or with IFN–γ (10 ng/mL) for 20 h, followed by immunoassay–based assessment [17] of OVA antagonistic peptide–driven IL–2 secretion from murine OVA–specific B3Z T cell hybridoma (gift of Dr. H. Shen, Houston Methodist Research Institute, Houston, TX, USA).

Statistics—Unless stated otherwise, findings are representative outcomes from three or more biological and technical replicates, using Student’s *t*-test for paired comparisons and ANOVA for group comparisons, as reported [6,9,14,15].

## 3. Results

### 3.1. Metabolomics Segregated Pirc and WT Rats According to Acute vs. Chronic SPI Intake

Pirc and WT rats fed basal AIN93 control (Ctrl) diet or AIN93 diet containing 10% *w*/*w* freeze–dried baby SPI for 26 weeks or 3 days (Figure 1A) were designated as AIN, SPI, and SPI3d groups, respectively. Among 17,243 metabolomic features identified in colon tissues at the end of the study, PLSDA segregated groups according to treatment and genotype (Figure 1B). For example, colon scrapings from WT rats (top right) and colon tumors from Pirc rats (lower left) had AIN and SPI3d groups clustered together, separate from SPI ‘chronic’ treatment. In other cases, namely, WT and Pirc normal ‘punch’ biopsies and Pirc colon scrapings, the SPI3d group segregated between AIN and SPI groups.

Five groups were distinguishable among the AIN controls, with Pirc tumor being segregated furthest from WT normal colon (Figure 1B, lower right). ‘Heatmaps’ averaged across the replicates in each group produced a distinct tumor metabolomic signature—especially in rats given SPI for 26 weeks (Figure 1C, top right). In correlation analyses, Pirc tumor was distinct from other groups, most notably for metabolomic features with increased relative abundance (Figure 1D, lower right). Thus, the metabolomic signature from chronic SPI treatment was distinct from AIN and SPI3d groups, especially for Pirc colon tumors.

### 3.2. Fatty Acids and Other Compound Categories Were Altered by SPI Intake

**Figure 2:**
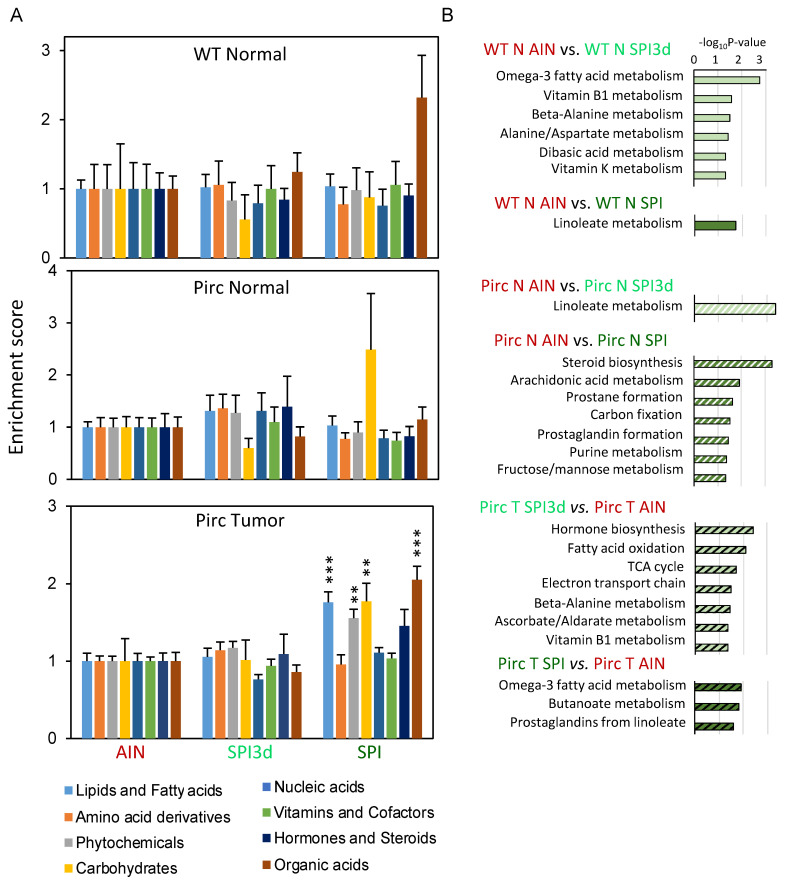
Compound categories prioritized from metabolomic analyses in Pirc and WT rats. (**A**) Mummichog coupled to KEGG Compound library identified eight categories of small molecules. Data are shown as mean ± SD. One–way ANOVA was used to compare the mean of each column with the mean of every other column in the dataset, with Tukey correction for multiple comparisons (GraphPad Prism 9.0); ** *p* < 0.01; *** *p* < 0.001. (**B**) Enriched metabolic pathways for the group comparisons indicated; mummichog cutoff at 0.2, *p* < 0.05 significance level. N, normal; T, tumor.

Mummichog coupled to KEGG prioritized eight categories of small molecules. In Pirc colon tumors, SPI increased four compound categories significantly, namely, Lipids and Fatty acids, Phytochemicals, Carbohydrates, and Organic acids (Figure 2A, lower right). This was not observed for SPI3d in Pirc tumors, and no statistically significant changes were detected in normal tissues from Pirc or WT rats. In pairwise comparisons, Omega–3 fatty acid metabolism, Butanoate metabolism, and Prostaglandins from linoleate were implicated in Pirc T SPI vs. Pirc T AIN groups (Figure 2B, bottom). Linoleate metabolism also featured significantly for WT N AIN vs. WT N SPI and Pirc N AIN vs. Pirc N SPI3d pairwise comparisons. Thus, among other changes, linoleate and butanoate metabolism were altered markedly by SPI intake in the rat.

**Figure 3:**
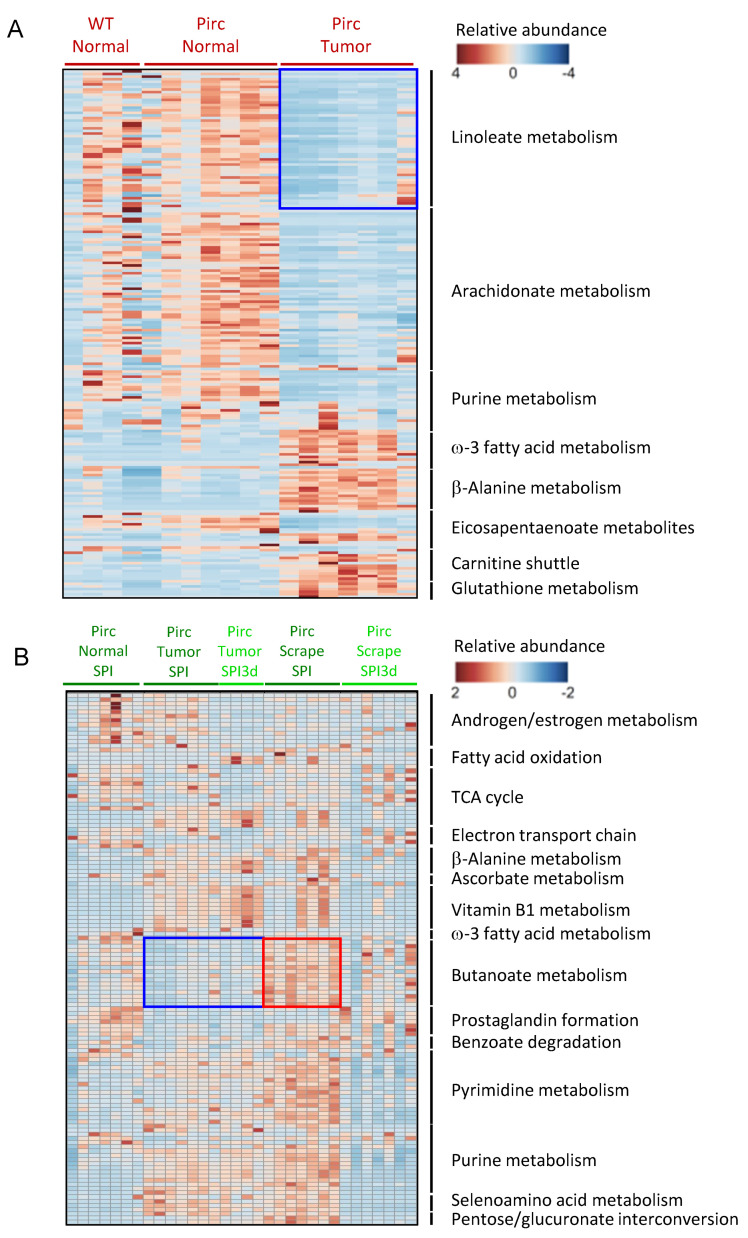
Heatmaps of enriched metabolomic pathways. (**A**) In the absence of SPI treatment, a Pirc tumor signature had reduced relative abundance of Linoleate metabolism, among other changes. (**B**) In SPI–treated rats, but not SPI3d, Pirc colonic scrapings had increased Butanoate metabolism, whereas tumors had a low corresponding abundance, among other changes. Each column represents the metabolomic profile of an individual animal in the corresponding group (n = 4–7 rats).

In metabolomic heatmaps, AIN group comparisons revealed a Pirc tumor signature that was distinct from Pirc normal and WT normal (Figure 3A). Noteworthy in tumors was the lower Linoleate metabolism in six out of seven rats (Figure 3A, blue square). Reduced Arachidonate, Purine, and Eicosapentaenoate metabolism and increased Carnitine shuttle and ω-3 fatty acid, β-Alanine, and Glutathione metabolism also was detected in tumors. In SPI and SPI3d groups, tumors had a lower relative abundance of Butanoate metabolism (Figure 3B, blue square). In the Pirc scrape SPI dataset there was increased Butanoate, Purine, Pyrimidine, and Selenoamino acid metabolism (Figure 3B, red square). We inferred that butanoate and other metabolites were increased by chronic SPI but not SPI3d in the zone closest to the colonic crypts, captured by colonic scraping.

**Figure 4:**
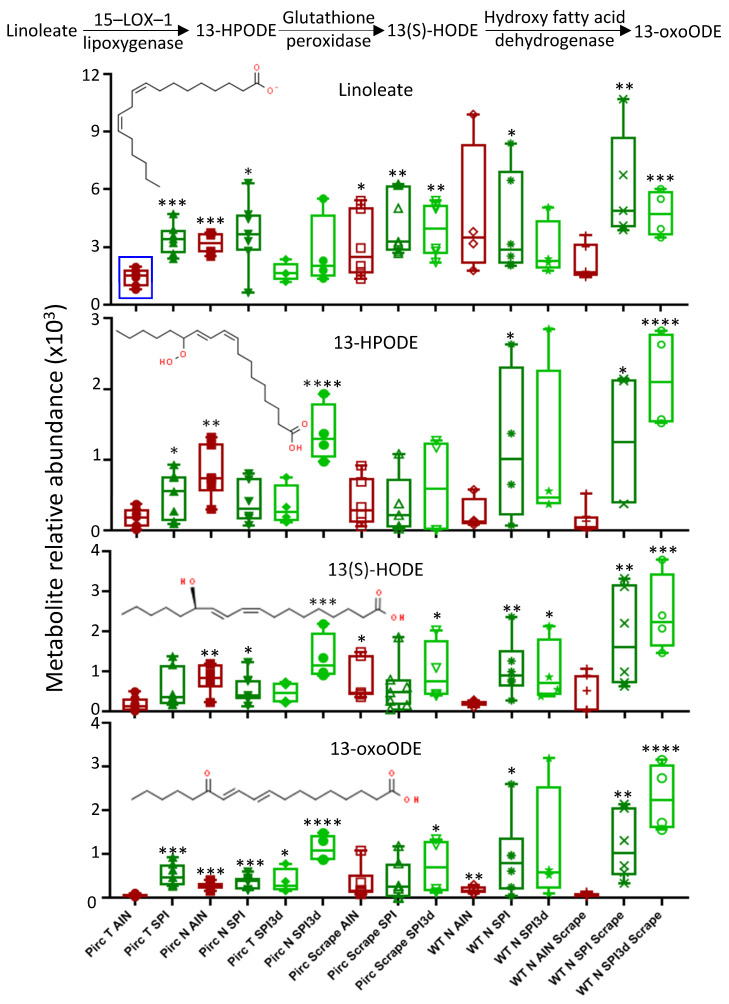
Linoleate and metabolites in rat colon tissues. Datapoints represent individual rats as biological replicates (n = 4–7). One–way ANOVA with Tukey correction; * *p* < 0.05; ** *p* < 0.01; *** *p* < 0.001; **** *p* < 0.0001. 13–HPODE, 13–hydroperoxy–9Z,11E–octadecadienoic acid; 13(S)–HODE, (13S)–hydroxyoctadecadienoic acid; 13–oxoODE, (9Z,11E)–13–oxooctadeca–9,11–dienoic acid.

Low linoleate metabolism in colon tumors of Pirc AIN controls (Figure 3A, blue square) was taken for further analyses (Figure 4, top left, blue square). Groups from our prior work [6] are reproduced here as the first four bars in each dataset, with additional comparisons not reported previously. From the y–axis ranges in Figure 4, linoleate was present at ~3–4–fold higher levels than its 15–lipoxygenase–1/15-LOX-1 metabolites. Linoleate and its 15-LOX-1 metabolites had a lower relative abundance in Pirc T AIN controls (red bars), which was reversed or ‘normalized’ in the SPI and SPI3d groups. Exceptions were noted, however, including higher relative metabolite abundances in Pirc normal colon (third bar in each dataset). In Pirc tissues, the highest 15–LOX–1 metabolite levels were detected in SPI3d normal colon scrapings, as exemplified by 13(S)–HODE (Figure 4, *** *p* < 0.001). In general, the highest 15–LOX–1 metabolite abundances were for WT rats in SPI and SPI3d groups (Figure 4, datasets at right side). For example, 13(S)–HODE in WT N SPI3d scrape was significantly higher than in WT N AIN scrape (*** *p* < 0.001), as well as Pirc T AIN. We concluded that SPI treatment for 3 days or 26 weeks markedly increased linoleate and its 15–LOX–1 pathway intermediates in Pirc and WT colon tissues, including 13(S)–HODE. These observations are noteworthy given the proapoptotic antitumor activity reported for 13(S)–HODE in colorectal cancer [18,19,20,21,22,23,24].

### 3.3. Butanoate Metabolites and 13(S)–HODE Inhibited HDAC Activity

**Figure 5:**
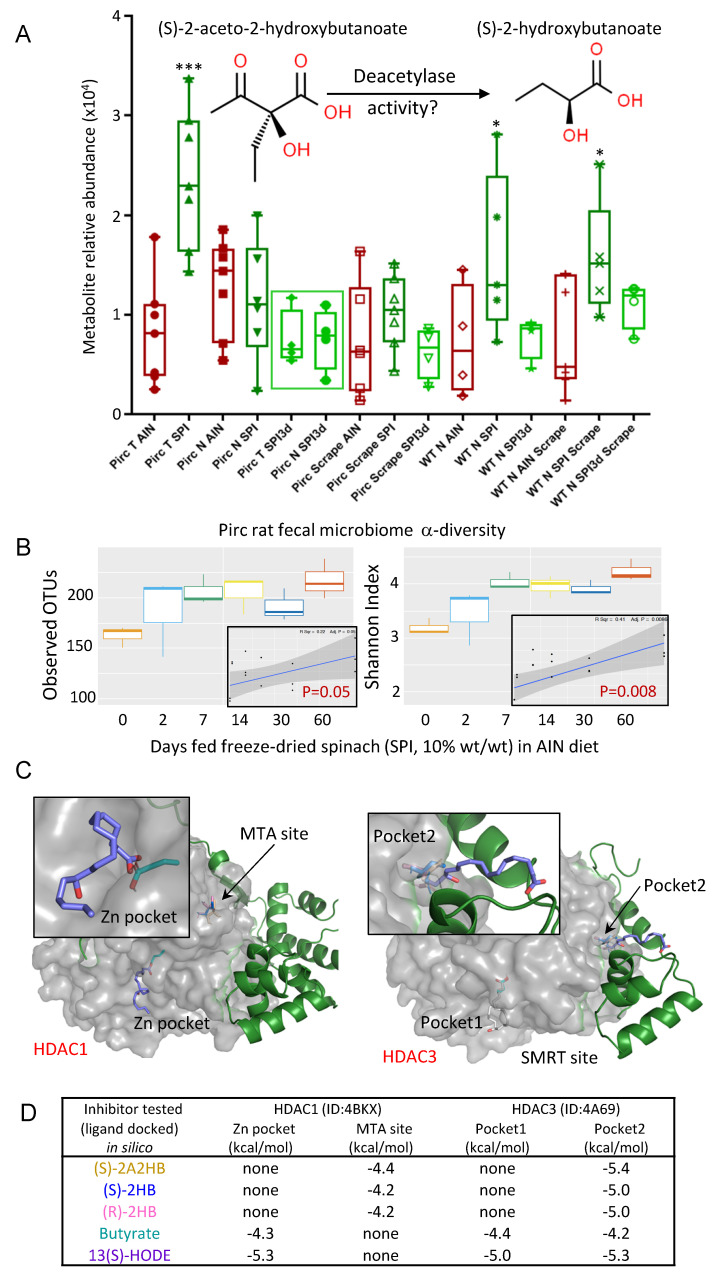
Butanoate metabolites exhibit favorable HDAC interactions. (**A**) Relative abundance of (S)–2–aceto–2–hydroxybutanoate from metabolomic analyses of colon tissues from Pirc and WT rats. One–way ANOVA with Tukey correction; *** *p* < 0.001; * *p* < 0.05. Also shown is the hypothetical generation of (S)–2–hydroxybutanoate ((S)–2HB) via cellular deacetylases. (**B**) Time–course of gut microbiome α-diversity following SPI intake. (**C**) Docking of butanoate metabolites and 13(S)–HODE with HDAC1 and HDAC3 complexes in silico. (**D**) Typical docking scores from n = 3 tests.

A metabolite linked to butanoate metabolism also was identified (Figure 5A), namely, (S)–2–aceto–2–hydroxybutanoate ((S)–2A2HB). Long–term SPI consumption increased (S)–2A2HB levels significantly in colon tumors compared to adenomatous polyps from AIN controls, and compared to Pirc normal tissues ± SPI intake (*** *p* < 0.001). Interestingly, SPI3d had no such effect (Figure 5A, green square). These trends were recapitulated in WT N SPI and WT N SPI scraping samples vs. SPI3d and the corresponding WT AIN controls (Figure 5A). The findings hinted at insufficient time for reshaping of the gut microbiome by SPI3d in order to fully enhance butyrate–producing bacteria. Using reported methodologies in the Pirc model [6], a time–course study revealed that increased α-diversity required 7–14 days of dietary SPI intake, and plateaued thereafter (Figure 5B).

A limitation of the current investigation was the inability to secure (S)–2A2HB from reputable commercial sources, to validate target tissue concentrations. However, we hypothesized that cellular deacetylases might generate localized (S)–2–hydroxybutanoate ((S)–2HB) from (S)–2A2HB (Figure 5A), akin to the mechanism–based HDAC inhibition by sulforaphane metabolites [8]. Using reported methodologies for molecular docking in silico [10,14], favorable interactions were predicted for (S)–2A2HB, (S)–2HB and the enantiomeric metabolite (R)–2HB with allosteric sites in HDAC1– and HDAC3–containing corepressor complexes (Figure 5C). 13(S)–HODE interacted with both allosteric sites in HDAC3 and with the zinc–containing catalytic site of HDAC1, resembling the known HDAC1/HDAC3 inhibitor butyrate [25,26,27,28]. Docking scores were in the range –4.2 to –5.4 kcal/mol (Figure 5D), synonymous with reversible inhibition.

**Figure 6:**
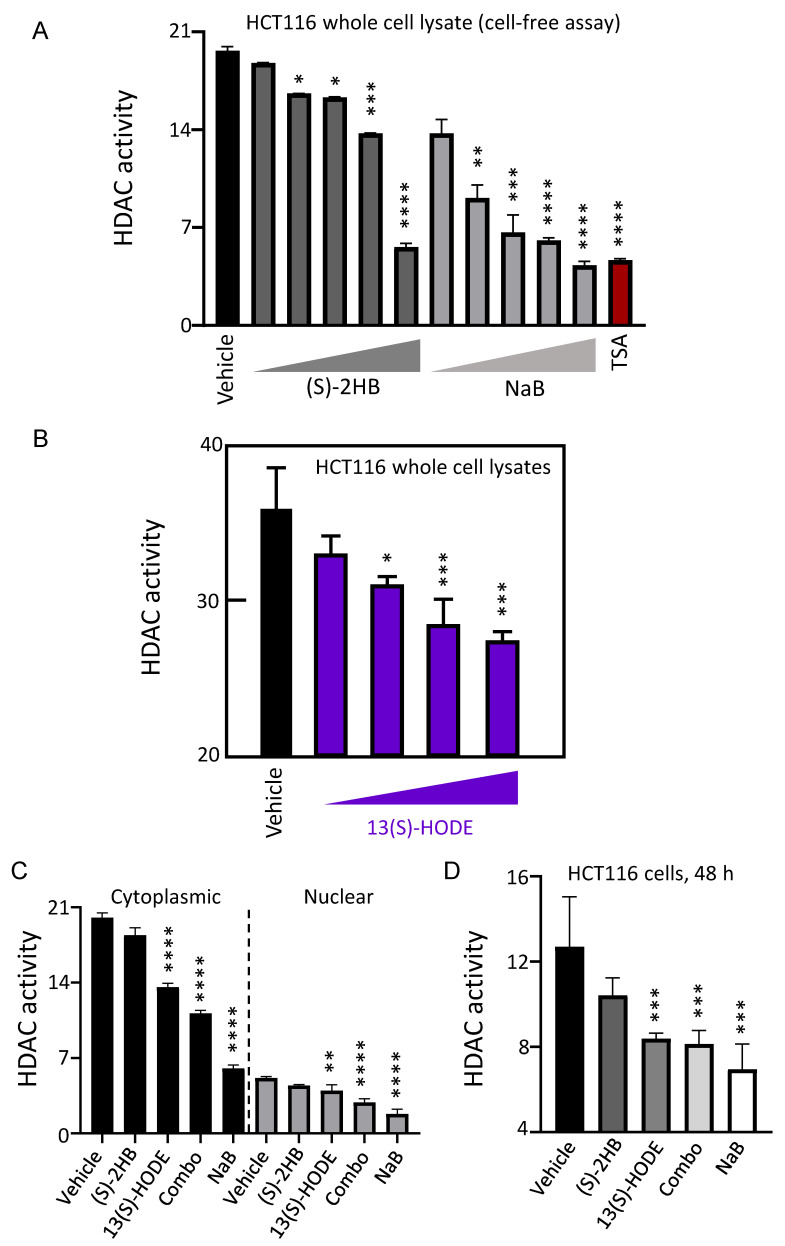
(S)–2HB and 13(S)–HODE are HDAC inhibitors. (**A**) Inhibition of HDAC activity in HCT116 whole cell lysates incubated with (S)–2HB or sodium butyrate (NaB); wedge symbol, 62.5, 125, 250, 500 and 1000 μM; Trichostatin A (TSA) (0.1 μM). (**B**) HDAC inhibition in HCT116 whole cell lysates incubated with 0.625, 1.25, 2.5 and 5 μM 13(S)–HODE. (**C**) HDAC inhibition in cytoplasmic and nuclear extracts from HCT116 cells treated with 100 μM (S)–2HB, 2.5 μM 13(S)–HODE, 100 μM (S)–2HB + 2.5 μM 13(S)–HODE (Combo), or 1 mM NaB. (**D**) HCT116 cells were treated for 48 h with agents at concentrations used in (**C**), and whole cell lysates were tested for HDAC activity. Mean ± SD, n = 3, * *p* < 0.05; ** *p* < 0.01; *** *p* < 0.001; **** *p* < 0.0001. Y–axis, relative fluorescence units × 10^4^.

Butyrate can attain millimolar concentrations in the gut [25,26,27,28], but information often is lacking for its metabolites. Test compounds were screened using a reported HDAC activity assay [8,9,10], with Trichostatin A (TSA) as a positive control in some experiments. In a cell–free assay with whole cell lysates from HCT116 human colon cancer cells, concentration–dependent inhibition of HDAC activity was observed by (S)–2HB and sodium butyrate (NaB) at 62.5, 125, 250, 500 and 1000 μM (Figure 6A), and by 0.625, 1.25, 2.5 and 5 μM 13(S)–HODE (Figure 6B). Cytoplasmic and nuclear lysates from HCT116 cells also were treated with selected inhibitor doses. Compared to vehicle control, 2.5 μM 13(S)–HODE alone or in combination with 100 μM (S)–2HB inhibited HDAC activity significantly, similar to 1 mM NaB (Figure 6C). Thus, deacetylase activities in nuclear and non–nuclear compartments were susceptible to inhibitor treatments. Analogous results were obtained when HCT116 cells were incubated with test agents for 48 h and the whole cell lysates were added to HDAC activity assays (Figure 6D).

### 3.4. Apoptosis Induction Was Observed by 13(S)–HODE ± (S)–2HB

**Figure 7:**
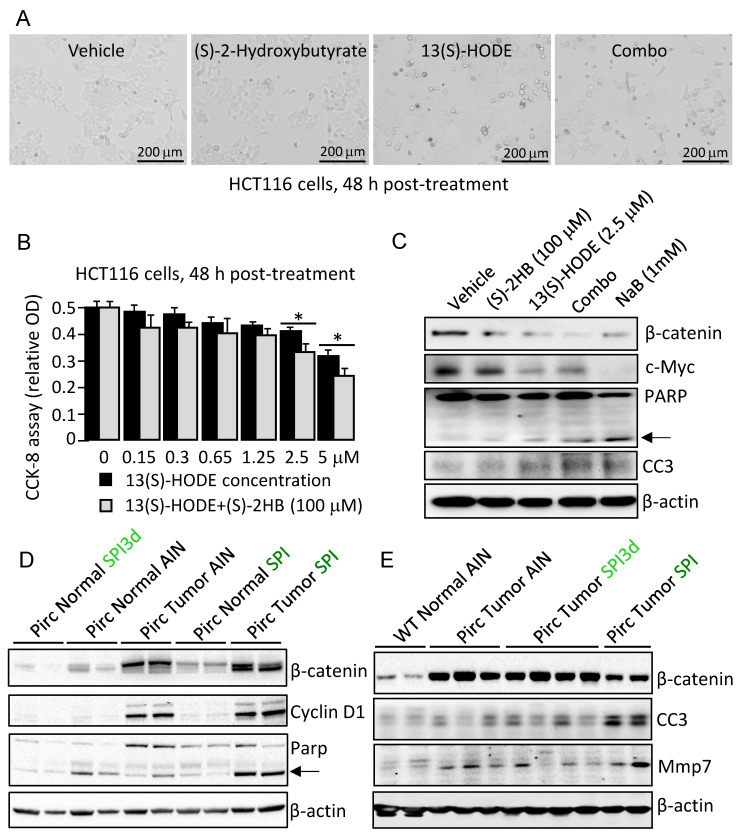
Apoptosis induction is observed by 13(S)–HODE, (S)–2HB, and spinach. (**A**) Morphology, (**B**) viability (mean ± SD, n = 3, * *p* < 0.05 by Student’s *t*-test) and (**C**) apoptosis endpoints in HCT116 cells treated for 48 h with 100 μM (S)–2HB, 2.5 μM 13(S)–HODE, or 100 μM (S)–2HB plus 2.5 μM 13(S)–HODE (Combo). (**D**,**E**) Immunoblotting of Pirc and WT tissues, with β-actin as loading control. Each lane represents an individual tumor or normal tissue. Representative of findings from two or more independent experiments. CC3, cleaved (active) Caspase–3; arrow, cleaved PARP.

Based on published reports for 13(S)–HODE concentrations in cell–based assays [18,19,20,21,22,23,24] and for (S)–2HB from the HDAC activity experiments (Section 3.3, above), HCT116 cells were treated with (S)–2HB, 13(S)–HODE, or the combination. At 48 h, phenotypic readouts included cell rounding/detachment, decreased cell viability, and cleaved PARP and Caspase–3 (Figure 7A–C). The threshold concentration for apoptosis induction in HCT116 cells at 48 h was 100 μM for (S)–2HB and 2.5 μM for 13(S)–HODE. Combined at these concentrations, 13(S)–HODE+(S)–2HB were comparable to 1 mM NaB in terms of apoptosis induction and loss of β-catenin or c–Myc protein expression (Figure 7C). Similar observations were made with aqueous and organic extracts of spinach added to human colon cancer cells (data not shown).

In vivo, β-catenin, Cyclin D1 and Mmp7 clearly designated tumor from normal colon; however, no downregulation of Wnt/β-catenin targets was observed with dietary SPI intake (Figure 7D,E). Interestingly, SPI but not SPI3d increased cleaved PARP and Caspase–3 levels markedly in Pirc colon tumors. Thus, apoptosis induction could be uncoupled from β-catenin downregulation in vivo, and other mechanisms were pursued.

### 3.5. 13(S)–HODE and (S)–2HB Targeted the IFN-γ Signaling Axis

**Figure 8:**
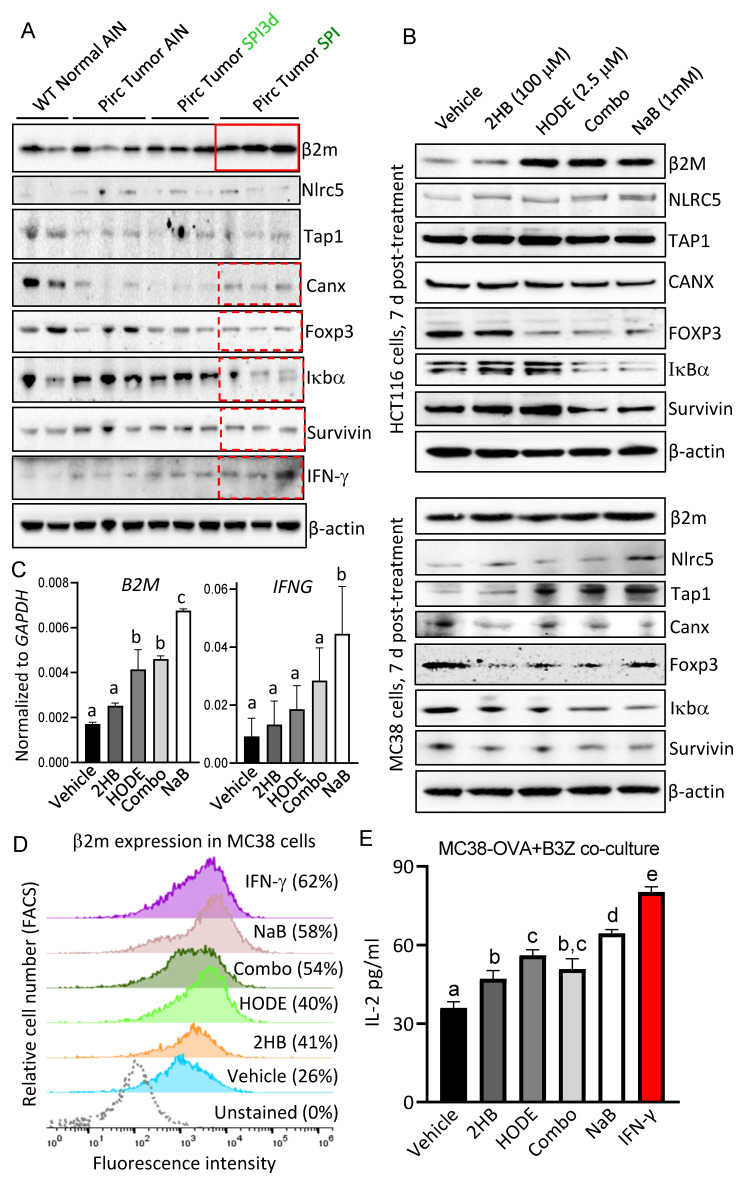
13(S)-HODE and (S)-2HB target the IFN-γ signaling axis. (**A**) Immunoblotting of Pirc and WT tissues. Each lane represents an individual tumor or normal tissue. (**B**) Immunoblotting of HCT116 and MC38 cells 7 d after treatment with 100 μM (S)-2HB, 2.5 μM 13(S)-HODE, or 100 μM (S)-2HB plus 2.5 μM 13(S)-HODE, with 1 mM NaB as a control. (**C**) *B2M* and *IFNG* mRNA expression in HCT116 cells following 7-d treatments as in ‘B’, RT-qPCR data normalized to *GAPDH*; mean ± SD, n = 3. (**D**) FACS-based analysis of β2m cell suface occupancy in MC38 cells 7 d after treatment with test agents as in ‘B’, or with IFN-γ (10 ng/mL) for 20 h. (**E**) Immunoassay-based assessment [17] of ovalbumin (OVA) antagonistic peptide-driven IL-2 secretion from murine OVA-specific B3Z T cell hybridoma co-incubated with MC38-OVA cells [16], treated as in panel ‘C’. Mean ± SD, n = 3. Bars with different superscripts differed significantly based on group comparisons via one-way ANOVA.

Transcriptomics previously identified IFN-γ signaling as a priority in the SPI-treated rat [6]. As a working hypothesis, HDAC inhibition by linoleate and butanoate metabolites might activate components of the IFN-γ signaling axis, including epigenetically–silenced major histocompatibility complex class I (MHC–I) factors [29,30,31,32]. Immunoblots (Figure 8A) confirmed upregulation of β2m and IFN-γ in Pirc colon tumors following chronic SPI intake, as compared to WT normal AIN, Pirc tumor AIN, and Pirc tumor SPI3d groups. No corresponding changes were noted for Nlrc5, a master transcriptional regulator of MHC–I signaling [29], or endoplasmic reticulum transporters such as Tap1. Calnexin (Canx), a chaperone protein involved in the folding of MHC–I molecules [33], was markedly downregulated in Pirc colon tumors, and chronic SPI intake partially reversed this trend (Figure 8A, dashed red box)—although not to the levels observed in WT normal AIN controls. Compared to Pirc tumor AIN and Pirc tumor SPI3d groups, Pirc tumor SPI samples had reduced Foxp3, Iκbα, and Survivin expression (Figure 8A).

Human HCT116 and murine MC38 colon carcinoma cells were incubated for 7 days with (S)–2HB±13(S)–HODE or NaB at concentrations used previously (Figure 7C), and immune–related targets were assessed via immunoblotting. In HCT116 cells (Figure 8B, top) increased expression of β2M and NLRC5 coincided with decreased FOXP3, IκBα and Survivin, especially for NaB and (S)–2HB+13(S)–HODE combination treatment. Similar observations were made for these molecular targets in MC38 cells, and for Tap1 induction (Figure 8B, bottom). After 7 days of treatment, RT–qPCR data in HCT116 cells revealed increased mRNA expression for targets such as *B2M* and *IFNG* in the relative order: (S)–2HB < 13(S)–HODE < (S)–2HB+13(S)–HODE (Combo) < NaB (Figure 8C). A similar order of efficacy was detected in FACS–based experiments that assessed cell surface occupancy of β2m in MC38 cells, i.e., vehicle 26%, (S)–2HB 41%, 13(S)–HODE 40%, (S)–2HB+13(S)–HODE 54%, NaB 58% and IFN-γ 62% (Figure 8D). Finally, immunoassay–based assessment of ovalbumin (OVA) antagonistic peptide (SIINFEKL)–driven IL–2 secretion from murine OVA–specific B3Z T cell hybridoma co–incubated with MC38–OVA cells corroborated the functionality of MHC–I complexes at the cell surface, in the relative order: (S)–2HB < 13(S)–HODE ≤ (S)–2HB+13(S)–HODE < NaB < IFN-γ (Figure 8E). Thus, components of the IFN-γ signaling axis were confirmed as mechanistic targets of linoleate and butanoate metabolites in cell–based assays, and in the colon tumors from rats fed SPI on a chronic basis, but not SPI3d.

## 4. Discussion

Recent human clinical intervention trials have assessed diverse aspects of SPI intake, including anthropometric measures, muscle fitness, metabolic profiles, arterial stiffness, and urinary biomarkers [5,34,35,36], extending prior research on the health benefits of green leafy vegetables and the functional properties of spinach–derived phytochemicals and bioactives [37,38,39,40,41]. We reported that long–term feeding of freeze–dried SPI at 10% *w*/*w* in the diet for 26 weeks exhibited significant antitumor efficacy in the Pirc model, resulting in >60% reduced tumor multiplicity in the colon and small intestine [6]. In Apc–mutant and WT rats, increased gut microbiome diversity after SPI consumption coincided with reversal of taxonomic composition. Metagenomic prediction implicated linoleate and butanoate metabolism, tricarboxylic acid cycle, and pathways in cancer, which was supported by transcriptomics and metabolomics. Thus, tumor suppression by SPI involved marked reshaping of the gut microbiome and changes in host RNA–miRNA networks. When colon polyps were compared with matched normal–looking tissues via metabolomics, anticancer outcomes were linked to SPI–derived linoleate bioactives with known anti–inflammatory/proapoptotic mechanisms in colorectal cancer [18,19,20,21,22,23,24].

The current investigation confirmed and extended these observations, and sought to compare long–term vs. acute (26–week vs. 3d) SPI consumption in the rat, incorporating colonic mucosa scrapings and tissues from WT animals. Partial least squares discriminant analyses of 17,243 metabolomic features aligned SPI3d with AIN controls, or distributed SPI3d midway between AIN and SPI groups. These findings hinted at SPI3d starting to reshape metabolomic features towards the more marked changes observed after 26 weeks of SPI intake. Heatmaps revealed a distinct Pirc colon tumor metabolomic signature, with SPI (but not SPI3d) increasing lipids and fatty acids, organic acids, carbohydrates, and phytochemicals. Based on our prior report [6], we focused initially on linoleate and its downstream metabolites. Interestingly, 13–HPODE, 13(S)–HODE and 13–oxoODE had some of the highest relative abundance levels in WT tissues, especially from colon scrape samples in SPI and SPI3d groups (Figure 4). This implicated SPI–derived (rather than tumor–specific) linoleate metabolites, consistent with their detection in the freeze–dried baby SPI incorporated into AIN basal diet, using unbiased metabolomic analyses [6].

Interestingly, among the 700+ metabolomic features in baby SPI, no (S)–2A2HB was detected [6]. The significant increase in (S)–2A2HB in Pirc colon tumors by SPI and its absence following SPI3d treatment (Figure 5A, green square) suggested the necessity for reshaping of the gut microbiome over several weeks, to increase α–diversity and enhance butyrate–producing bacteria. This was confirmed in a time–course investigation, in which α–diversity increased in the Pirc model after approximately 7–14 days of dietary SPI intake, and plateaued thereafter for up to 60 days (Figure 5B).

As noted above, (S)–2A2HB was not available commercially, but the presumed deacetylated metabolite (S)–2HB was viewed as a possible mechanism–based HDAC inhibitor, analogous to sulforaphane metabolites [8,34]. Using molecular docking in silico, favorable interactions were predicted for (S)–2A2HB, (S)–2HB and the enantiomeric metabolite (R)–2HB with allosteric sites in HDAC1– and HDAC3–containing corepressor complexes (Figure 5C). Notably, 13(S)–HODE also had favorable interactions with HDAC3 allosteric sites and with the catalytic pocket of HDAC1. Docking scores were in the range –4.2 to –5.4 kcal/mol (Figure 5D), synonymous with the degree of HDAC inhibition detected in cell–free and cell–based HDAC activity assays, relative to 0.1 μM TSA. Tissues in the Pirc model were prioritized for metabolomics, but future work should examine changes in selected HDACs and histone acetylation or methylation marks following SPI and SPI3d intake.

In human colon cancer cells, a threshold was observed for apoptosis induction at 48 h using 100 μM (S)–2HB and 2.5 μM 13(S)–HODE. When combined, these metabolites caused marked induction of cleaved PARP and Caspase–3, comparable to 1 mM NaB. Decreased viability in cell–based assays coincided with loss of β-catenin, but this was not recapitulated in vivo. Thus, expression of β-catenin, Cyclin D1, and Mmp7 remained high in adenomatous polyps compared to normal colon, and was unaffected by SPI3d or SPI treatment, although SPI increased cleaved PARP and Caspase–3 in Pirc colon tumors, indicative of apoptosis induction. Quantifying nuclear ‘active’ β-catenin might yield greater insights, as described in recent studies that provided genetic and molecular corroboration for specific LOX enzymes and linoleic metabolites in suppressing LPR5 recycling, Wnt/β-catenin signaling, and colon carcinogenesis [24].

The IFN-γ signaling axis was defined as a top priority in Pirc SPI colon tumors at 30 weeks [6]. In the current investigation, Pirc tumor SPI samples had increased expression of β2m, interferon-γ and Canx as compared to Pirc tumor SPI3d and Pirc tumor AIN groups, and decreased levels of Foxp3, Iκbα and Survivin, implicating NFκB signaling and apoptosis induction. Several of the immune biomarkers were similarly altered in cell–based assays involving human and murine colon carcinoma cells incubated with (S)–2HB±13(S)–HODE. Notably, increased cell surface occupancy of β2m was confirmed in FACS–based analyses, and the functionality of MHC–I complexes was corroborated in MC38–OVA+B3Z co–culture experiments. Downregulation of MHC–I components is a potential oncogenic driver [29,30,31,32,33], and the targeting of epigenetic ‘readers’, ‘writers’ and ‘erasers’ might facilitate re–expression of cell surface MHC complexes to reengage host immune pathways in cancer cells. These mechanisms also might be pertinent at earlier stages, as in the case of adenomatous colon tumors from the Pirc model and in FAP or Lynch Syndrome patients, which harbor predicted MHC neoantigens [42]. Llosa et al. [43] noted that ‘an altered amino acid due to a coding mutation is only relevant as a tumor neoantigen for T cells if it can be processed and presented on self–MHC…individual tumors with lower mutational load can nonetheless generate good T–cell neoepitopes if the mutations are appropriately positioned’. A roadblock to appropriately positioned neoepitopes involves epigenetic silencing of MHC components, and the ability of linoleate and butanoate metabolites to inhibit HDAC activity and to re–express MHC functional complexes at the surface of colon cancer cells is worthy of further investigation. 

## 5. Conclusions

This investigation compared long–term vs. acute SPI intake in a preclinical model of hereditary colon cancer, and corroborated our prior findings vis–à–vis SPI–derived linoleate bioactives and butanoate metabolites linked to increased α-diversity of the gut microbiome. This is the first report to demonstrate HDAC inhibition, apoptosis induction, and altered IFN-γ signaling in colon cancer cells treated with specific butanoate and linoleate metabolites in combination. Future work should seek to corroborate the concentrations of these metabolites in vivo in the context of apoptosis induction in colon tumors after long–term dietary SPI intake, and phenotypic outcomes following treatment of FAP patient organoids. Clinical translation of freeze–dried whole foods, such as SPI, to at–risk patients might provide valuable quality–of–life benefits via inflammasome/immune mechanisms, delaying colectomy and drug intervention [44,45,46,47,48,49,50].

## Data Availability

Multi–omics data associated with this article were deposited in GEO with accession number GSE180160.
